# A Case of Idiopathic Twenty-Nail Dystrophy

**DOI:** 10.7759/cureus.70037

**Published:** 2024-09-23

**Authors:** Radha Kumar, Ashikabanu Mujibur Rahman, Navin Umapathy, Shami Kumar, Vaanmathi Azhagar Nambi Santhi

**Affiliations:** 1 Paediatrics, Saveetha Medical College and Hospital, Saveetha Institute of Medical and Technical Sciences, Saveetha University, Chennai, IND

**Keywords:** alopecia areata, linear ridges, self-limiting, trachyonychia, twenty nail dystrophy

## Abstract

In children, nail diseases can be either congenital or acquired, with an occurrence rate of 3 to 11% in the pediatric population. In both fingers and toes, rough, accentuated linear ridges of the nails are referred to as trachyonychia. This condition most commonly occurs in childhood, with a higher prevalence in males. Twenty-nail dystrophy refers to a disorder affecting all twenty nails. While the cause is idiopathic, it may also be associated with other dermatological conditions. The disorder typically affects the nails on both hands and feet in a bilateral and symmetrical pattern. The primary concern for the child is usually cosmetic disfigurement, requiring regular follow-up and long-term care.

In this case report, we describe a 10-year-old boy who presented with a six-year history of twenty-nail dystrophy, with no significant family history of skin or nail diseases. The child exhibited no other symptoms aside from alopecia areata. This condition is self-limiting and is managed symptomatically. This case report aims to highlight the importance of physical examination in the early diagnosis and treatment of nail disorders.

## Introduction

Twenty-nail dystrophy, also called trachyonychia, originates from the Greek word 'trakos,' which means rough. It was first identified in 1950 by Alkiewicz, and later in 1977 by Hazelrigg, it was termed the twenty-nail dystrophy of childhood. This condition is an acquired, idiopathic nail dystrophy that affects all twenty nails [1]. Although the exact prevalence is unknown, various studies estimate an incidence ranging from 3 to 11% [2]. It typically occurs in infancy or childhood and often persists into adulthood; it is a benign condition [3]. The most common age of onset is between three and twelve years, with males being more frequently affected [4]. Studies done by Lee YB et al. revealed that childhood-onset trachyonychia more commonly occurs in males, whereas adult-onset trachyonychia has female predominance [5]. This disorder may be associated with other skin or systemic diseases, most commonly with alopecia areata, lichen planus, and psoriasis.

There are two distinct clinical types of trachyonychia: opaque and shiny trachyonychia. Opaque trachyonychia is the more common and more severe type, characterized by thickened nails with longitudinal ridges and a sand-papered appearance, whereas shiny trachyonychia is the less severe type, characterized by uniform, shiny nails with pits that reflect light. In opaque trachyonychia, nail changes are caused by a persistent, fluctuating inflammatory assault on the nail matrix that continues without stopping. In contrast, shiny trachyonychia involves an intermittent, localized, and regularly recurring inflammatory attack on the matrix, with intervals of normal matrix function. Of these, shiny trachyonychia is most commonly associated with alopecia areata [2,6]. A thorough examination is necessary to identify any associated treatable conditions. Several studies in the literature indicate that this condition is self-limiting, making reassurance for both parents and children essential. Some studies have revealed that the use of short-term (4 to 6 months) topical corticosteroids for those who are bothered and whose quality of life is affected.

## Case presentation

**Figure 1:**
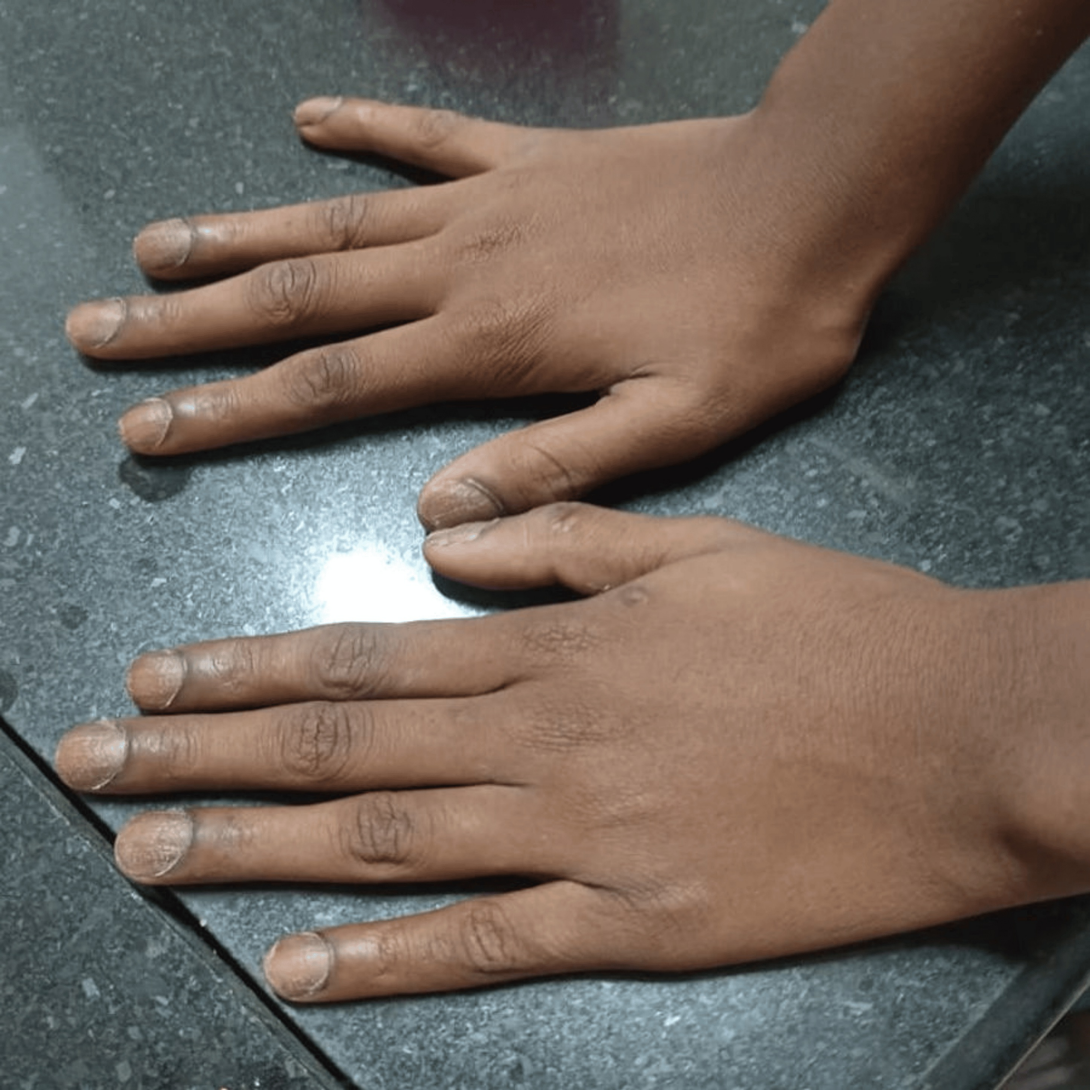
Discoloration, dryness, and dystrophy of hand nails.

**Figure 2:**
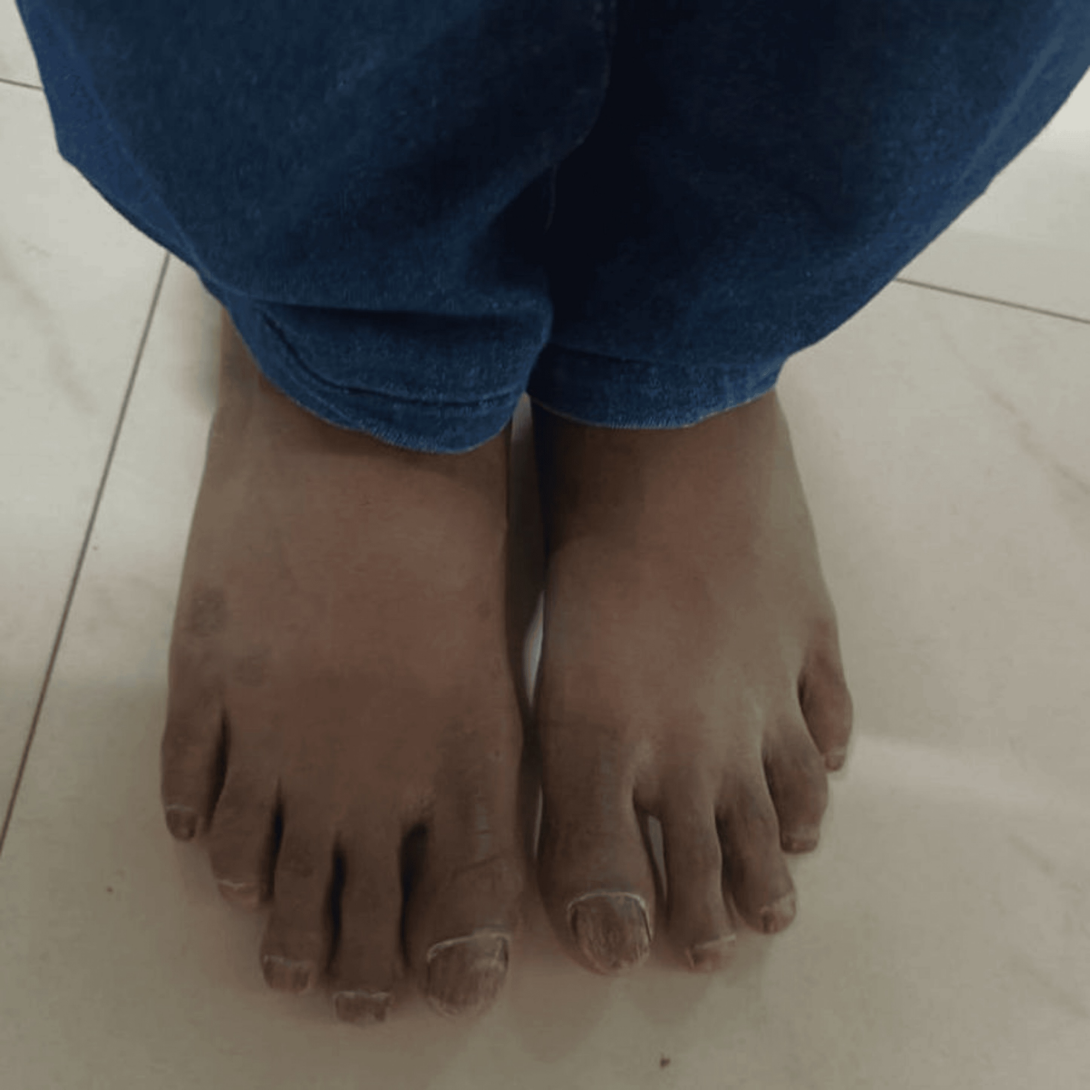
Dry, brittle, and ridged nails on the feet.

**Figure 3:**
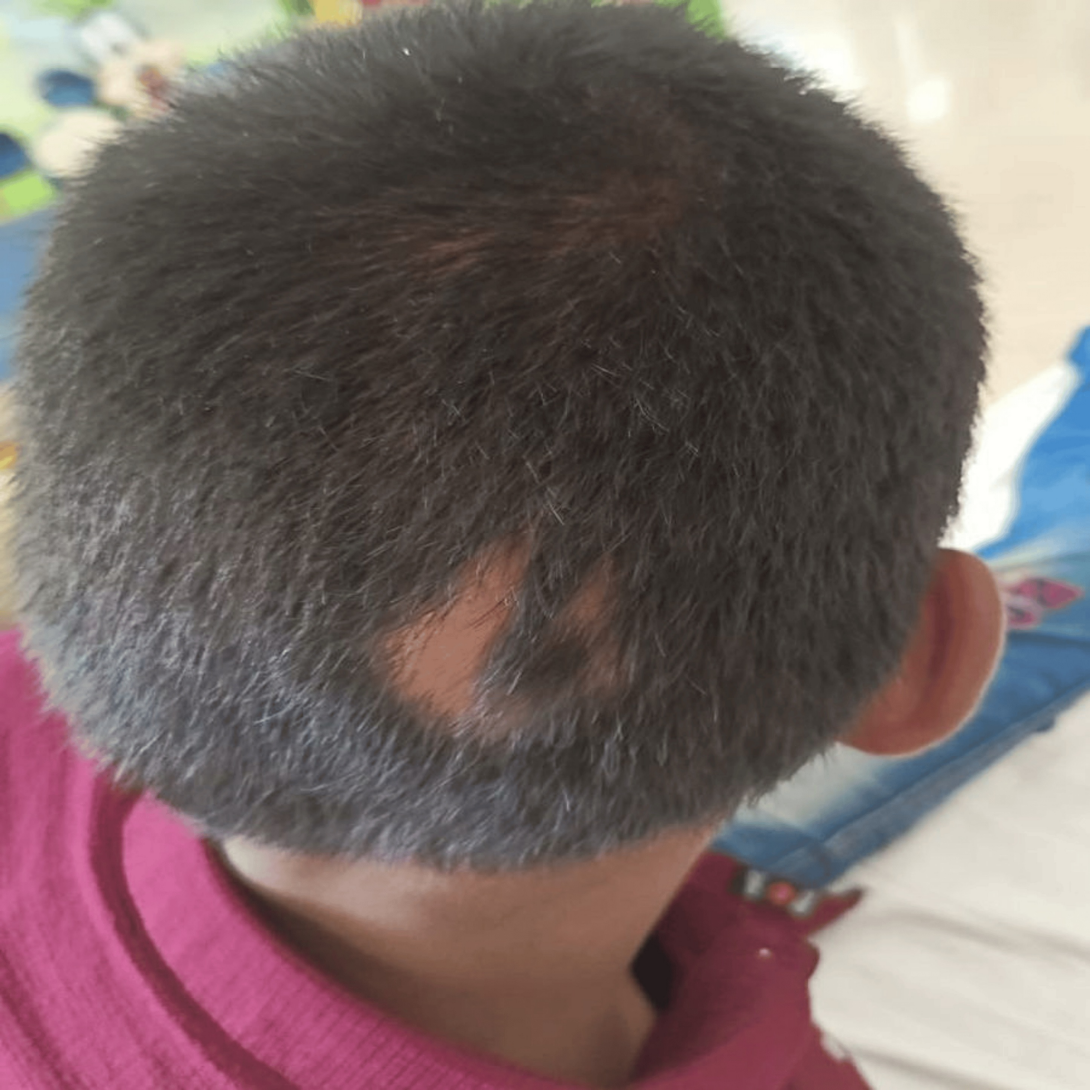
Localized alopecia areata in the occipital region of the head.

A 10-year-old boy, born to parents in a non-consanguineous marriage, presented with complaints of a six-year history of loss of texture and normal lustre involving fingers and toes. Initially, the child’s little finger was affected, then the condition gradually progressed to affect all nails on both hands and feet, which were discolored and rough. Initially, the child had taken some Ayurvedic treatment with no response. There was an associated history of patchy hair loss over the occipital region noted for the past three years. The child also complained of dry skin, but there were no skin lesions elsewhere on the body. There was no history of redness, rash, or itching over the skin. There was no previous history of allergies, and no history of skin or nail diseases in the family. A physical examination of the child’s nails revealed discolored, thickened, dystrophic nails with longitudinal ridges and pitting, which involved the nails of both hands (Figure 1) and feet (Figure 2), with localized alopecia areata present over the occipital region of the head (Figure 3). The systemic examination was normal.

Nail scrapings tested negative for pathogenic fungi (Table 1), and routine blood tests returned normal results. The child had no other complaints other than cosmetic discomfort.

**Table 1:** Nail scraping report shows negative results for pathogenic fungi. KOH: Potassium hydroxide.

40% KOH	No fungal elements seen/visualized
Fungal culture	No fungal growth at 5 days of incubation/ No fungus grown in culture after 5 days of incubation.

A diagnosis of idiopathic trachyonychia was made after consulting with a dermatologist, as all twenty nails were affected and there were no systemic symptoms. The child was started on tacrolimus for nails and minoxidil 5% for alopecia areata. Both the parents and the child were reassured, and he was on regular follow-up. On follow-up, minimal hair growth was present.

## Discussion

‘Twenty-nail dystrophy,’ also known as trachyonychia, affects all twenty nails and is characterized by a lack of luster and diffuse ridging of the sandpaper-like surface of nails in the most severe cases. In some instances, nail plate abnormality is less severe, presenting with small, numerous, superficial pits that give the nails a shiny appearance [6,7]. Nails are typically thin and brittle, with pronounced longitudinal ridging, resulting in an opaque and rough appearance of the nail plate in trachyonychia, along with a hyperkeratotic and ragged appearance of the cuticle. This condition can occur either as an idiopathic disorder without any cutaneous or systemic findings or be associated with various other disorders like alopecia areata, psoriasis, lichen planus, incontinentia pigmenti, vitiligo, and congenital cutaneous candidiasis. It is autosomal dominant in some families, with monozygotic twins also being affected, as reported in some literature, and can appear in multigenerational families [8]. It is associated with lichen planus in 4 to 18.5% of patients, psoriasis in 13 to 26%, alopecia areata in 45 to 83%, atopic dermatitis, and ichthyosis vulgaris [9,10]. Among these, alopecia areata is the most commonly associated disease, more commonly seen in the shiny type of trachyonychia. Alopecia areata is a complex autoimmune condition that causes nonscarring alopecia of either a diffuse or local form [11]. A complete physical examination of the nail and a nail matrix biopsy can help to establish the cause. While a nail biopsy is not routinely performed in all cases of idiopathic trachyonychia, histopathologic features show focal spongiotic inflammation of the nail matrix. Studies done by Tosti A et al. revealed that histopathologic features of idiopathic trachyonychia showed spongiotic changes and features of psoriasis, lichen planus, and pemphigus vulgaris, and trachyonychia associated with alopecia areata showed both spongiotic changes and features of lichen planus [2,12].

Differential diagnoses include brittle nails, psoriasis, and lichen planus. In lichen planus, longitudinal fissures and pterygium can occur, which are absent in trachyonychia, while alopecia areata features geometric pitting that resembles shiny trachyonychia. It is primarily a self-resolving disease, most commonly affecting children. Parents are often very anxious and eager to seek medical advice. It is crucial to counsel them on the nature and progression of the disease, as reassurance plays a key role in managing the condition [3].

Pharmacological treatments include intralesional injections of triamcinolone at a dose of 2.5 to 3 mg/ml, administered into the nail folds of the proximal region. These injections are painful and very difficult to administer in children. Systemic involvement may require treatment with medications such as biotin, prednisolone, etretinate, and antimalarials. Topical psoralen and ultraviolet A (UVA) light therapy over a seven-month course have also been reported to be effective. Additionally, to improve the appearance of the nails, clear nail hardeners can be applied. Intensive treatment with cyclosporine A at a dose of 3 mg/kg/day may also be used [13]. Another commonly employed treatment is biotin; one study reported the successful use of biotin at a dosage of 20 mg per day in treating twenty-nail dystrophy in two patients with primary biliary cirrhosis. In approximately 50% of cases, nail changes show significant improvement and resolve completely without leaving scars within five to six years of treatment [14]. When determining the optimal treatment plan for a patient with idiopathic trachyonychia, it is important to consider the patient's age, the subtype of trachyonychia, the severity of the condition, the patient’s previous history of any treatments taken for trachyonychia, and any associated dermatologic and non-dermatologic diseases.

## Conclusions

Idiopathic twenty-nail dystrophy was diagnosed, accompanied by alopecia areata, and was treated conservatively with tacrolimus and 5% minoxidil. On follow-up, the child showed improvement in hair growth. The specific cause of the inflammation affecting the nail unit in trachyonychia patients remains uncertain. This is a chronic clinical condition that can occur on its own or in association with various dermatologic or non-dermatologic conditions, such as sarcoidosis, amyloidosis, and immunoglobulin A deficiency. Although the condition resolves independently, treatment should be provided only when necessary. It is important to reassure both the child and the parents to prevent ineffective or harmful medical treatments. Ongoing follow-up is essential, as idiopathic trachyonychia can sometimes precede the development of other cutaneous diseases.

## References

[1] Shanmuganathan H, Kumar R (2019). A case of twenty nail dystrophy affecting a 12-year-old boy. Int J Contemp Pediatr.

[2] Tosti A, Bardazzi F, Piraccini BM, Fanti PA (1994). Idiopathic trachyonychia (twenty-nail dystrophy): a pathological study of 23 patients. Br J Dermatol.

[3] Sehgal VN (2007). Twenty nail dystrophy trachyonychia: an overview. J Dermatol.

[4] Dehesa L, Tosti A (2012). Treatment of inflammatory nail disorders. Dermatol Ther.

[5] Lee YB, Cheon MS, Park HJ, Cho BK (2012). Clinical study of twenty-nail dystrophy in Korea. Int J Dermatol.

[6] Haber JS, Chairatchaneeboon M, Rubin AI (2017). Trachyonychia: review and update on clinical aspects, histology, and therapy. Skin Appendage Disord.

[7] Robert LB (2001). Baran & Dawber's diseases of the nails and their management. J Cosmet Dermatol.

[8] Commens CA (1988). Twenty nail dystrophy in identical twins. Pediatr Dermatol.

[9] Silverman RA, Rhodes AR (1984). Twenty-nail dystrophy of childhood: a sign of localized lichen planus. Pediatr Dermatol.

[10] Taniguchi S, Kutsuna H, Tani Y (1995). Twenty nail dystrophy (trachyonychia) caused by lichen planus in a patient with alopecia universalis and ichthyosis vulgaris. J Am Acad Dermatol.

[11] Darwin E, Hirt PA, Fertig R, Doliner B, Delcanto G, Jimenez JJ (2018). Alopecia areata: review of epidemiology, clinical features, pathogenesis, and new treatment options. Int J Trichology.

[12] Tosti A, Fanti PA, Morelli R (1991). Trachyonychia associated with alopecia areata: a clinical and pathologic study. J Am Acad Dermatol.

[13] Ohta Y, Katsuoka K (1997). A case report of twenty-nail dystrophy. J Dermatol.

[14] Chu DH, Rubin AI (2014). Diagnosis and management of nail disorders in children. Pediatr Clin North Am.

